# Proteome Characteristics of Non-Alcoholic Steatohepatitis Liver Tissue and Associated Hepatocellular Carcinomas

**DOI:** 10.3390/ijms18020434

**Published:** 2017-02-17

**Authors:** Anna Kakehashi, Vasily E. Stefanov, Naomi Ishii, Takahiro Okuno, Hideki Fujii, Kazuaki Kawai, Norifumi Kawada, Hideki Wanibuchi

**Affiliations:** 1 Department of Molecular Pathology, Osaka City University Graduate School of Medicine, Asahi-machi 1-4-3, Abeno-ku, Osaka 545-8585, Japan; m1159070@med.osaka-cu.ac.jp (N.I.); m2026860@med.osaka-cu.ac.jp (T.O.); wani@med.osaka-cu.ac.jp (H.W.); 2 Department of Biochemistry, Saint Petersburg State University, Saint Petersburg 199034, Russia; vastef@mail.ru; 3 Department of Hepatology, Osaka City University Graduate School of Medicine, Osaka 545-8585, Japan; rolahideki@med.osaka-cu.ac.jp (H.F.); kawadanori@med.osaka-cu.ac.jp (N.K.); 4 Department of Environmental Oncology, Institute of Industrial Ecological Sciences, University of Occupational and Environmental Health, Kitakyushu 807-8555, Japan; kkawai@med.uoeh-u.ac.jp

**Keywords:** nonalcoholic steatohepatitis (NASH), hepatocellular carcinoma, biopsy, PPARs, β-catenin, cytokeratins 8/18, NRIP1, oxidative stress

## Abstract

To uncover mechanisms of nonalcoholic steatohepatitis (NASH) associated hepatocarcinogenesis, we compared the proteomes of human NASH-associated liver biopsies, resected hepatocellular carcinomas (HCCs) and HCCs of HCV^+^ patients with normal liver tissue of patients with gastrointestinal tumor metastasis, in formalin-fixed paraffin-embedded samples obtained after surgery in our hospital during the period from 2006 to 2011. In addition, proteome analysis of liver tumors in male STAM NASH-model mice was performed. Similar changes in the proteome spectrum such as overexpression of enzymes involved in lipid, cholesterol and bile acid biosynthesis and examples associated with suppression of fatty acid oxidation and catabolism, alcohol metabolism, mitochondrial function as well as low expression levels of cytokeratins 8 and 18 were observed in both human NASH biopsies and NASH HCCs, but not HCV^+^ HCCs. Alterations in downstream protein expression pointed to significant activation of transforming growth factor β, SMAD family member 3, β-catenin, Nrf2, SREBP-LXRα and nuclear receptor-interacting protein 1 (NRIP1), and inhibition of PPARs and p53 in human NASH biopsies and/or HCCs, suggesting their involvement in accumulation of lipids, development of fibrosis, oxidative stress, cell proliferation and suppression of apoptosis in NASH hepatocarcinogenesis. In STAM mice, PPARs inhibition was not obvious, while expression of cytokeratins 8 and 18 was elevated, indicative of essential differences between human and mouse NASH pathogenesis.

## 1. Introduction

Non-alcoholic fatty liver disease (NAFLD), which has become a very common disease with the prevailing increase in obesity across the world, is generally considered to feature two stages—fatty liver (FL) (non-alcoholic fatty liver (NAFL)) followed in some cases by non-alcoholic steatohepatitis (NASH). However, no specific test is presently available to predict progression of NAFL to NASH. Fatty liver is generally recognized as a benign and non-progressive condition [1,2]. NASH is now widely known as a liver disease with the potential to cause cirrhosis, and finally hepatocellular carcinoma (HCC), although recent evidence has suggested that NAFLD may directly promote hepatic carcinogenesis independent of cirrhosis [3]. People with NAFLD do not necessarily have a history of excessive alcohol use. Many NASH patients are obese, but non-obese individuals maybe also affected by the disease. A genetic predisposition and metabolic factors are believed to be important in NASH pathogenesis [4]. It has been found that approximately 70% of diabetic people have some form of NAFLD, and that approximately 5% to 20% of people with diabetes have cirrhosis due to NASH, while the five-year HCC rate in NASH patients is 0%–15% [3,5,6]. While liver cirrhosis secondary to hepatitis C infection is still the main cause of liver cancer and the most common indication for liver transplantation in the world, the prevalence of NAFLD and NASH-related cirrhosis and HCC will become an increasingly major health care problem in the future. At present, different NAFLD therapies are being directed to improve insulin resistance (IR), but a really effective treatment does not exist [4].

Previous findings have indicated that neovascularization, which is significantly increased during the development of liver fibrosis, as well as oxidative stress, cytokeratin (CK) 18 expression, IR, tumor necrosis factor-α (TNF-α) and adiponectin status play pivotal roles in the progression of NASH to HCC [7,8]. IR, as a feature of obesity, increases the activity of hormone-sensitive lipase, which enhances lipolysis in visceral adipose tissue, thus resulting in the release of free fatty acids (FFAs) which are preferentially converted to triglycerides (TGs) within hepatocytes [8]. In addition, several studies have suggested that increased apoptosis of hepatocytes and angiogenesis have important roles in progression from simple FL to NASH, and correlate with disease severity and hepatic fibrosis [9,10]. Nevertheless, at present, the pathogenesis of NASH is still not well understood.

In the present study, results of liquid chromatography and tandem mass spectrometry (LC-Ms/Ms) and Ingenuity Pathway analysis (IPA) of NASH-associated human biopsies in a high fibrosis stage and resected HCCs were compared with protein spectra of HCCs of hepatitis C virus (HCV)-positive (HCV^+^) patients and normal-appearing liver tissue from cases with metastases from gastrointestinal cancers. Furthermore, a mouse NASH HCC model was employed for comparison of human and mouse NASH-related changes in the proteome, upstream regulators, molecular functions and signaling pathways.

## 2. Results

### 2.1. Proteome Analysis of Nonalcoholic Steatohepatitis (NASH) Biopsies, NASH-Associated and Hepatitis C Virus-Positive Hepatocellular Carcinomas

#### 2.1.1. Transforming Growth Factor β Signaling Pathway

The results of LC-Ms/Ms and IPA analyses of differentially expressed proteins in NASH biopsies, HCCs and HCV^+^ HCCs compared with normal-appearing liver tissue of patients with metastatic gastrointestinal tumors are presented in Table 1. Clinicopathological characteristics of NASH and NASH-associated and HCV^+^ HCC patients are presented in Supplementary Tables S1 and S2.

In all NASH-associated biopsies and HCCs, we observed significant elevation of numerous extracellular matrix and cytoskeleton proteins, the majority of them being downstream of transforming growth factor β (TGF-β), including different types of collagens, fibronectin (FN), intermediate filament vimentin (VIM), β actin-like protein 2 (ACTB2), myosin 9 (MYH9), tropomyosin α-4 chain (TPMA4), tubulin α-1C chain (TUBA1C), moesin (MSN), lumican (LUM) and others (Table 1). Furthermore, their elevation was more pronounced in NASH-associated HCCs as compared with HCV^+^ HCCs, likely due to the advanced cancer stages of NASH as compared to HCV^+^ patients. Interestingly, in contrast to the up-regulated proteins involved in development of fibrosis and stellate cell activation, expression of intermediate filament members cytokeratin 8 (CK8 (KRT8)) and cytokeratin 18 (CK18 (KRT18)) was significantly lower in all examined NASH-associated biopsies and HCCs, while in the majority of examined HCV^+^ HCCs (70%), we found significant increase in comparison to the normal-appearing liver tissue of patients with gastrointestinal tumor metastases.

#### 2.1.2. Proteins Involved in Lipid Metabolism and Formation of Oxidative Stress

Regarding altered expression of proteins associated with oxidative stress and its control in NASH biopsies and HCCs, in both cases superoxide dismutase [Mn], mitochondrial (SOD2) and thioredoxin (TXN) were significantly overexpressed, while catalase (CAT) was downregulated. In NASH HCCs, we further found significant elevation of cytochrome P450, family 51, subfamily A, polypeptide.1 (CYP51A1), cytochrome P450, family 4, subfamily F, polypeptide11 (CYP4F11) and cytochrome P450, family 8, subfamily B, polypeptide1 (CYP8B1), which are involved in lipid, cholesterol and bile acid biosynthesis, likely regulated through sterol regulatory element-binding proteins (SREBP)—liver X receptor α (LXRα) pathway. While proteins regulating lipid biogenesis were overexpressed in NASH HCCs, examples responsible for lipid catabolism, including molecules downstream of peroxisomal proliferating receptor γ (PPARγ) and peroxisome proliferating receptor α (PPARα), as well as other mitochondrial and peroxisomal proteins involved in process of oxidation of fatty acids, such as enoyl-CoA hydratase, mitochondrial (ECHS1), 3-ketoacyl-CoA thiolase, mitochondrial (ACAA2) and fatty acid binding protein 2 (FABP1), were reduced (Table 1). Furthermore, elevation of proteins involved in nuclear factor (erythroid-derived 2)-like 2 (NFE2L2; Nrf2)-mediated signaling and degradation of superoxide radicals, such as SOD2, was found to be associated with NASH pathogenesis.

#### 2.1.3. Mitochondrial Stress

In addition, in NASH-associated biopsies and HCCs, suppression of protein expression of numerous enzymes involved in control of mitochondrial function, the urea cycle (ornithine carbamoyltransferase (OTC), carbamoyl-phosphate synthase (CPS1)), amino acid metabolism (glutamate dehydrogenase 1 (GLUD1), aldehyde dehydrogenase family 4 member A1 (ALDH4A1), aspartate aminotransferase, mitochondrial (GOT2)), metabolism of ketone bodies (acetyl-CoA acetyltransferase (ACAT1), hydroxymethylglutaryl-CoA synthase (HMGCS2)) and alcohol metabolism (alcohol dehydrogenases 1B (ADH1B), 1C (ADH1C) and 4 (ADH4)) was found. Transcriptional factors prohibitin 1 (PHB1) and prohibitin 2 (PHB2), involved in control of mitochondrial respiration, function, cell proliferation and apoptosis, and related to induced oxidative stress conditions, were strongly overexpressed in NASH HCCs and moderately elevated in HCV^+^ HCCs, as compared to the normal liver tissue of metastatic tumor patients.

#### 2.1.4. Regulators of Cell Proliferation and Apoptosis

Next, we focused on alterations of expression of proteins regulating cell proliferation and cell death, and in particular, apoptosis in NASH-associated HCCs as compared to HCV^+^ HCCs. It was observed that expression of proteins downstream of β-catenin (cadherin-associated protein, β1) (CTNNB1) and CMYC, was strongly altered in NASH HCCs, indicating that both upstream regulators were significantly activated (Table 1). Furthermore, in NASH-associated HCCs, expression of a positive regulator of apoptosis (apoptosis-inducing factor 1, mitochondrial (AIFM1)) and annexin A6 (ANXA6) was suppressed. In contrast, protein levels of negative regulators of apoptosis (PHB1 and PHB2; cell death regulator Aven (AVEN)) were high, which might have been in response to the continuous oxidative stress associated with NASH. Furthermore, IPA results showed expression of downstream proteins of tumor protein p53 (TP53/p53) to be lower than in control tissue.

### 2.2. Altered Upstream Regulators Detected by Ingenuity Pathway Analysis (IPA)

**Figure 1:**
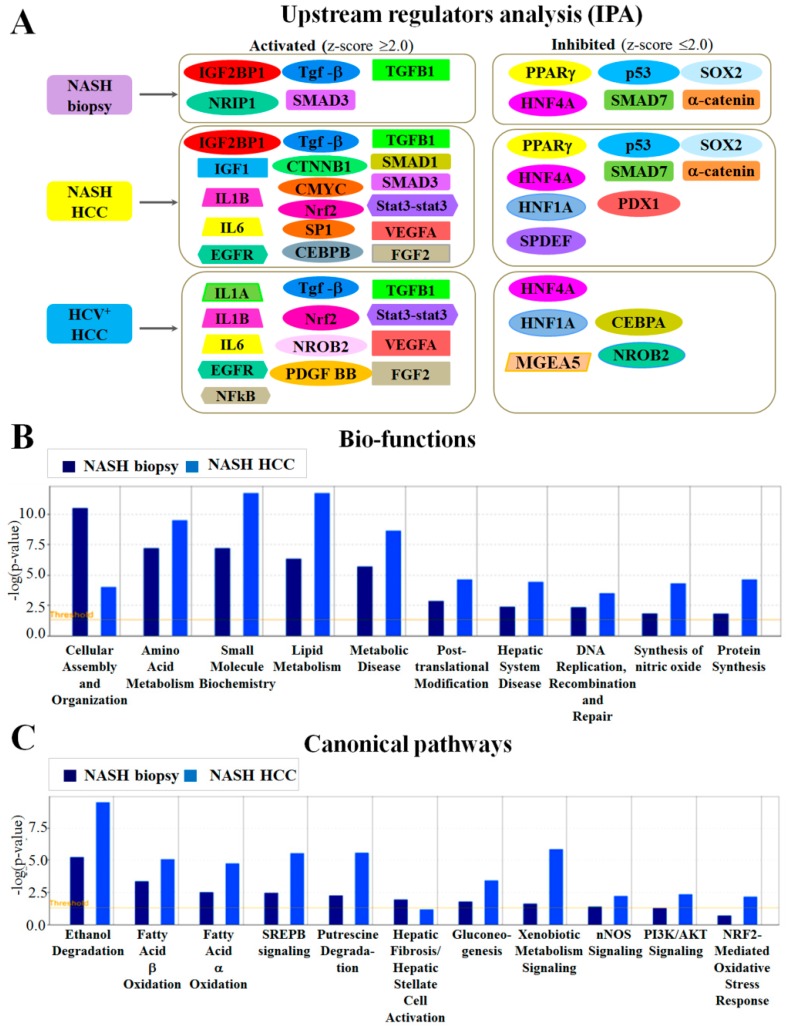
(**A**) Summary of comparative analysis of upstream regulators which activation (*z*-score ≥ 2.0) or inhibition (*z*-score ≤ 2.0) predicted by IPA; (**B**) functional; and (**C**) canonical pathways analyses of NASH-associated biopsies, HCCs and HCV^+^ HCCs.

Results of IPA upstream regulator analysis pointed to significant activation of the insulin-like growth factor 1 (IGF1) pathway and IGF2BP1 in NASH-associated HCCs, but not in HCV^+^ HCCs (Figure 1A and Supplementary Table S3). Furthermore, in NASH HCCs, IPA predicted significant activation (*z*-score ≥ 2.0) of TGF-β, transforming growth factor β1 (TGFB1), TGF-β receptor type 1 (TGFBR1) and type 2 (TGFBR2), SMAD1, SMAD3, Stat3-Stat3, CTNNB1 (β-catenin), CMYC, CCAAT/enhancer-binding protein β (CEBPβ), transcriptional factor Sp1 (SP1), nuclear factor κ-light-chain-binding protein (NFκB), interleukins β (IL1B) and 6 (IL6), NFE2L2 (Nrf2), vascular endothelial growth factor A (VEGFA), epidermal growth factor receptor (EGFR) and basic fibroblast growth factor 2 (FGF2).

Furthermore, significant suppression (*z*-score ≤ 2.0) of peroxisome proliferating receptor γ (PPARγ), α-catenin, SMAD7, HNF1A, NR1I3 (CAR), PXR ligand-PXR-retinoic acid-RXRα (PXR complex), TP53 (p53), SAM pointed domain-containing Ets transcription factor (SPDEF), transcription factor SOX-2 (SOX2), pancreas/duodenum homeobox protein 1 (PDX1) and a strong tendency for reduction of peroxisome proliferating receptor α (PPARα) were detected (Figure 1A and Table S3).

In NASH-associated biopsies, IPA predicted significant activation of translation regulators IGF2BP1, TGFB1, SMAD3 and significant inhibition of PPARγ (PPARG), α-catenin, SMAD7 and hepatocyte nuclear factor 4A (HNF4A), as in NASH HCCs. An interesting novel finding concerned predicted activation of nuclear receptor-interacting protein 1 (NRIP1/nuclear factor RIP140) in NASH biopsies (Figure 1A and Table S3). This protein modulates transcriptional activation by steroid receptors and controls the balance between fat accumulation and energy expenditure, thus is likely to play an important role in NASH progression.

In HCV^+^ HCCs, IPA predicted significant activation of TGF-β, TGFB1, Stat3-Stat3, Nrf2, NFκB, VEGFA, EGFR, FGF2, platelet-derived growth factor BB (PDGF-BB), interleukins α (IL1A), β (IL1B), 6 (IL6) and downregulation of HNF1A, HNF4A, CEBPA and PXR, with a similar trend for PPARs and CAR (Figure 1A and Table S3).

### 2.3. Functional and Canonical Pathway Analyses by IPA

Comparative analysis of protein functions by IPA indicated that in NASH-associated biopsies, HCCs and HCV^+^ HCCs, numerous proteins involved in cellular assembly and organization, cellular invasion and migration were differentially expressed (Figure 1B and Table S4). In NASH and HCV^+^ HCCs, proteins regulating cell survival, cell death and apoptosis and synthesis of nitric oxide were significantly altered. In NASH biopsies and HCCs, significant suppression of fatty acid oxidation and activation of exocytosis was detected (Figure 1B and Table S4).

Examination of altered canonical pathways by IPA demonstrated that many proteins with altered expression in NASH-associated biopsies and HCCs were related to fibrosis/hepatic stellate cell activation, lipid biogenesis, fatty acid β- and α-oxidation I, ethanol degradation II, and activation of Nrf2, SREBP, PI3K/AKT and nNOS signaling, gluconeogenesis and putrescine degradation III (Figure 1C and Table S5). In HCV^+^ HCCs, many proteins participating in fibrosis/hepatic stellate cell activation, lipid metabolism, LPS/IL-1-mediated inhibition of RXR function, actin cytoskeleton organization and activation of signaling by Rho family GTPases were altered.

### 2.4. Observed Correlations between Characteristic Proteins Expression in Human Biopsies and HCCs and Their Association with Clinicopathological Variables

The similarities of the protein spectrum of NASH biopsies and NASH HCC patients were detected with IPA; however, these proteome signatures were clearly different from that of HCV^+^ HCC patients. In all examined patients, increased body mass index (BMI) was negatively correlated with PPARγ activation score (*p* = 0.038), but positively correlated with NRIP1 (*p* < 0.0001). In addition, CK18 expression level was reversely correlated with PHB2 (*p* = 0.037) in NASH HCCs. In NASH biopsies, positive relationship between HDL (*p* < 0.0001), LDL (*p* < 0.0001) levels, hepatocyte ballooning (*p* = 0.004) and inflammation was detected.

In HCV^+^ HCC patients, increased CK8 (*p* = 0.001) and CK18 (*p* = 0.021) expression in HCC was associated with development of pM (M) metastasis. In addition, activation of β-catenin was associated with vessel invasion (*p* = 0.015).

### 2.5. Preneoplastic and Neoplastic Lesions Developing in STAM Mice

Results of the histopathological analysis in STAM mice are presented in (Table S6). The number of putative preneoplastic foci (PPFs) observed in STAM mice was 11.7 ± 9.6 per mouse We observed basophilic, vacuolated and mixed type (vacuolated and basophilic) PPFs. Incidences of hepatocellular adenomas (HCA) and HCC in mice at 17 weeks of age were 100% (7 mice) and 28.6% (2 mice), respectively, with multiplicities of 6.86 ± 8.67 and 0.43 ± 0.79 no./mouse. Most tumor cells observed in STAM mice contained lipid droplets and glycogen granules. HCC structure was heteromorphic, featuring both large and small tumor cells, oval cells and inflammatory cells.

### 2.6. Proteome Analysis of STAM Mice Hepatocellular Carcinomas (HCCs)

Results of proteome analysis of STAM mice HCCs in comparison with C57Bl/6N mouse normal liver are presented in Table 2.

Several differences were observed between NASH human and STAM mouse HCC proteomes. Firstly, CK8 and CK18 expression was elevated in mouse HCCs, while in human NASH HCCs these cytokeratins were significantly reduced. Secondly, significant (3.6-fold) up-regulation of glutathione S-transferase Mu 1 (GSTM1), the enzyme involved in glutathione metabolism, and 2.2-fold elevation of peroxisomal bifunctional enzyme (EHHADH), involved in fatty acid β-oxidation in peroxisomes, which is the downstream of PPARα was detected in STAM mice HCCs. In contrast, fatty acid β-oxidation in mitochondria was suppressed; thus, the PPARα-controlling hydroxymethylglutaryl-CoA synthase (HMGCS2), 3-ketoacyl-CoA thiolase (ACCA2) and fatty acid-binding protein (FABP1) were underexpressed.

### 2.7. Representative Results of β-Catenin, wnt1, PHB1 and PHB2 Immunohistochemistry in Human NASH-Associated and HCV^+^ HCCs

**Figure 2:**
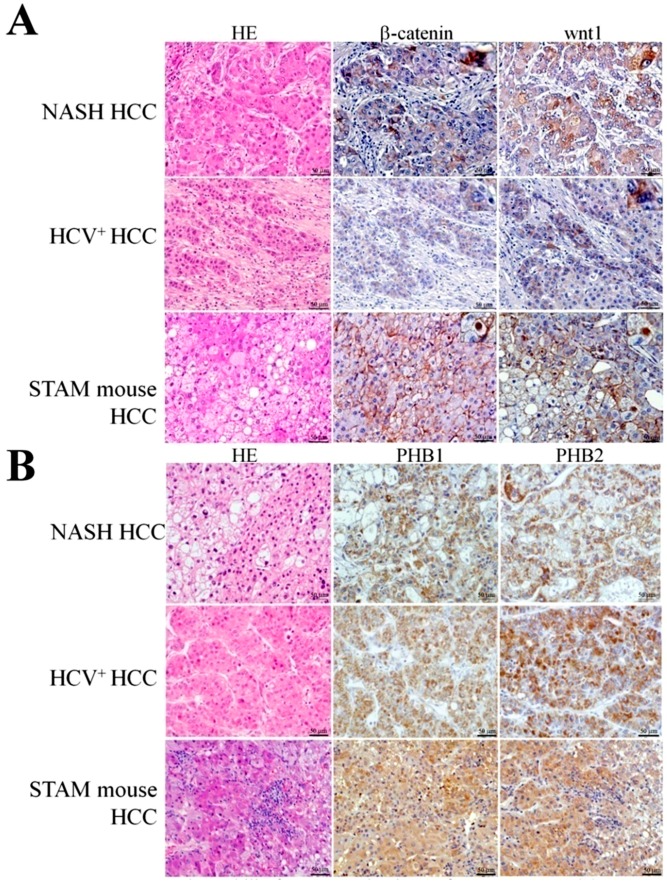

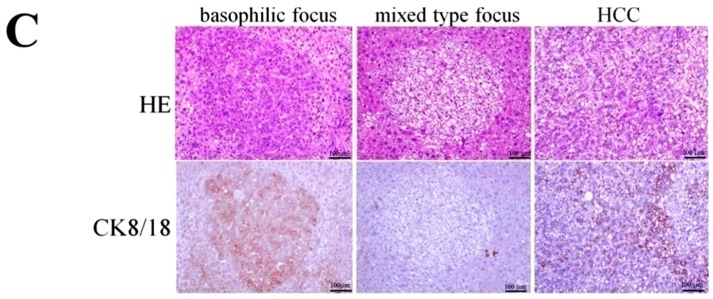
Histopathological and immunohistochemical analyses of human NASH-associated and HCV^+^ HCCs and STAM mice PPFs and HCCs: (**A**) immunohistochemical assessment of β-catenin and wnt1; (**B**) Immunohistochemistry for PHB1 and PHB2; and (**C**) immunohistochemistry for CK8/18 in the PPFs and HCCs STAM mice. Note the overexpression of β-catenin and wnt1 in the cell membrane, cytoplasm and/or nuclei of tumor cells in both human and STAM mouse HCCs.

Representative results of β-catenin and wnt1 immunohistochemical staining in human NASH-associated and HCV^+^ HCCs are presented in Figure 2A. In the specimens of NASH (10) and HCV^+^ HCC (10) patients examined, β-catenin was strongly elevated in the plasma membranes and cytoplasm of tumor, oval and bile ductul cells in four cases, and in plasma membrane and cytoplasm in three cases (40% and 30%, respectively; score 3+), moderately expressed in the plasma membrane and cytoplasm of four cases (40%; score 2+), weakly expressed in the plasma membrane and cytoplasm of one case (10%: score 1+) and negative in one case (10%; score 0). A strong correlation was found between β-catenin and wnt1 expression. Wnt1 was mostly overexpressed in the cytoplasm of tumor cells. In the livers of colorectal cancer patients, used as negative control for β-catenin and wnt1, no cytoplasmic β-catenin or wnt1 expression was observed in the normal-appearing liver cells. Only several positive ductal cells were present.

To confirm the results of proteome analysis, we further performed immunohistochemical assessment of PHB1 and PHB2 in NASH and HCV^+^ HCCs (Figure 2B). Strong positive expression of PHB1 and PHB2 (100%; score 2 or score 3) was observed in the cytoplasm of tumor cells in all NASH HCCs. In HCV^+^ liver cancers, cytoplasmic staining was found in seven of ten patients (70%; score 2 or score 3), while three (30%; score 0) were negative for both.

### 2.8. Immunohistochemical Assessment of β-Catenin and wnt1 in the Livers of STAM Mice

Representative results of β-catenin and wnt1 immunohistochemical staining in STAM mice HCCs is illustrated in Figure 2A. HCCs but not HCAs or PPFs of mice contained cells positive for β-catenin in the cytoplasm and/or nucleus. Positive expression of wnt1 was observed mainly in the cytoplasm of cancer cells. Tumor incorporated bile duct cell plasma membranes were positive for β-catenin. We also found numerous oval and ductal cells in mice HCCs positive for β-catenin and wnt1. In mouse tumors, the staining was mostly membranous, rarely cytoplasmic and nuclear, but in human HCCs rather cytoplasmic and nuclear. Observation of cytoplasmic and nuclear β-catenin expression indicated that translocation and activation of β-catenin occurred strongly in human and to the less extent in mouse NASH-associated HCCs.

### 2.9. Expression of Prohibitins and CK8/18 in STAM Mouse HCCs

Significant overexpression of both PHB1 and PHB2 was detected in the cytoplasm of HCC cells in STAM mice (Figure 2B). Basophilic PPFs were positive for CK8/18, however, mixed type and vacuolated foci contained only few CK8/18-positive cells (Figure 2C). CK8 and CK18 were overexpressed in STAM mice hepatocellular adenomas (HCAs) and HCCs with a basophilic pattern. However, in HCC regions with many incorporated lipid droplets, CK8/18 staining was less prominent as compared to the regions containing basophilic tumor cells (Figure 2C).

## 3. Discussion

**Figure 3:**
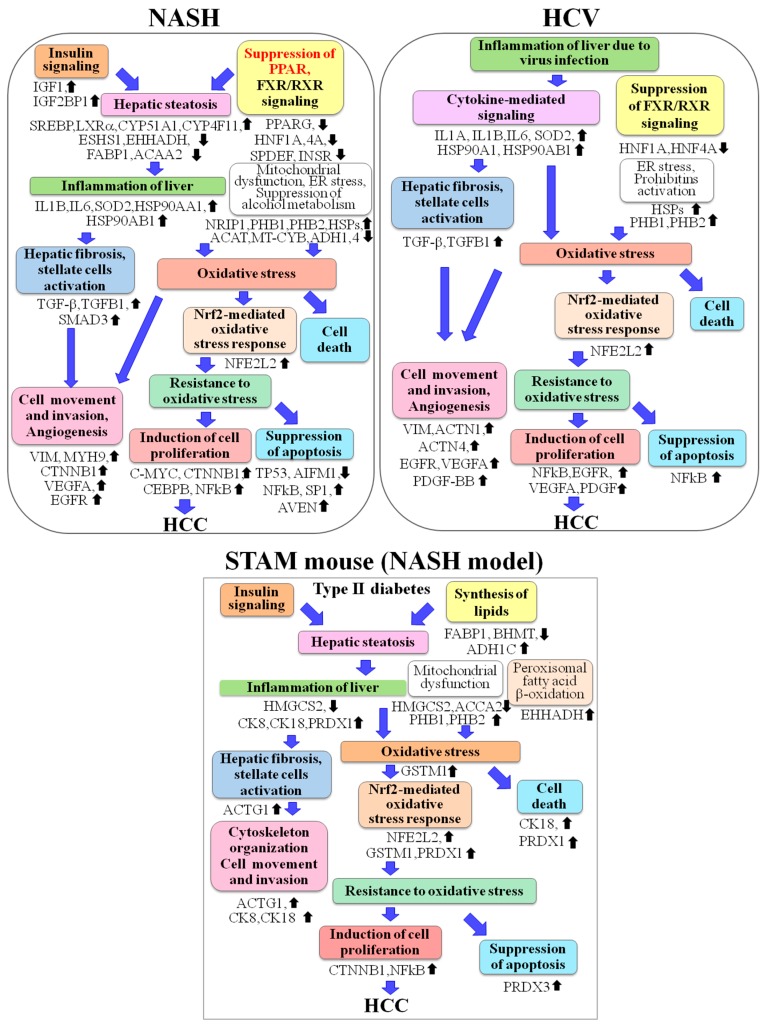
Events potentially contributing to NASH and Hepatitis C Virus-associated hepatocarcinogenicity. Black up and down arrows indicate increase/decrease in expression or activity for proteins or up-stream regulators.

Evaluation of proteome changes by QSTAR LC-Ms/Ms and IPA in NASH and HCV^+^ patients demonstrated numerous proteins with altered expression in NASH biopsies and HCCs known to be involved in fibrosis/hepatic stellate cell activation, lipid biogenesis, suppression of fatty acid β- and α-oxidation and ethanol degradation. Activation of IGF1, TGF-β and Wnt/β-catenin signaling, NRIP1, Nrf2, SREBP-LXRα, PI3K/AKT, CMYC, CEBPβ, transcriptional factor SP1, NFκB, IL1B and IL6, VEGFA, EGF, FGF2 and other upstream regulators, and downregulation of PPARs and p53 was predicted to be associated with NASH (Figure 3). It is conceivable that in NASH HCCs overexpression of enzymes involved in energy metabolism, extracellular matrix proteins, vimentin, actin cytoskeleton members, and those involved in DNA repair of double strand breaks and oxidative base modifications, is likely to be mediated by TGF-β, β-catenin, Nrf2, SP1, EGFR, PDGF, FGF2, VEGFA, IL1A, IL1B and other factors, which may explain lowered apoptosis and increased survival, migration and invasion activity of NASH liver cells. PHBs also are likely to play an important role in the TGF-β-mediated mesenchymal phenotype [11,12]. From these data, we conclude that along with IR and activation of TGF-β, an important role in NASH pathogenesis is played by dysregulation of Wnt/β-catenin, NRIP1, Nrf2, SREBP-LXRα, PHBs, PPARs and p53.

In previous studies, a “two-hit” theory has been suggested for the pathogenesis of NAFLD [13]. Accumulation of triglycerides as lipid droplets in cytoplasm of more than 5% of hepatocytes (steatosis) has been considered as the first hit, with IR reportedly contributing to this process. The first hit affects the liver making it susceptible to the second hit, which may feature development of oxidative stress (reactive oxygen species (ROS) generation), peroxidation of cardiolipin in inner mitochondrial membranes and mitochondrial dysfunction, production of pro-inflammatory cytokines, increase of apoptosis and gut-derived bacterial endotoxinemia, and thus processing to a necroinflammatory stage, finally defined as NASH. Our results in general support the existing theory, especially the conclusions concerning IR, mitochondrial dysfunction and development of oxidative stress. The results of IPA upstream regulator analysis showed significant activation of IGF1 signaling in NASH-associated HCCs, which was not found in HCV^+^ HCCs. Previously, diabetes has been reported to be associated with more severe NAFLD, but at the same time NAFLD worsen diabetes and promotes systemic inflammation processes in the liver [14]. Persistent hyperglycemia or glucotoxicity and IR are known to play a crucial role in development of hepatic steatosis by influencing both adipose tissue and hepatic metabolism. IR increases lipolysis and fatty acid flow from adipose tissue to the liver by impairment of the hormone-sensitive lipase in adipocytes. Furthermore, elevated glucose levels and hyperinsulinemia stimulate de novo lipogenesis in the liver by activation and up-regulation of lipogenesis transcriptional factors, including SREBP-1c and CREBβ [15]. In our study, most of the results were in line with previously reported data concerning activation of these two transcriptional factors. Thus, the newly established up-regulation of the cytochrome P450 superfamily of enzymes CYP51A1, 4F11 and 8B1 was likely to be downstream of SREBP-1c. CYP4F11 is known to be involved in the synthesis of cholesterol, steroids and other lipids and has been further shown to ω-hydroxylate long chain fatty acids which are then metabolized by alcohol dehydrogenase, aldehyde dehydrogenase, and dicarboxylyl CoA synthetase to form their respective Coenzyme A (CoA)-bound dicarboxylic acids [16]. Fatty acids are then transferred either to peroxisomes, where they undergo shortening and conversion to phospholipids, triglycerides, and cholesterol esters, or to mitochondria for complete β-oxidation [17]. If expression of CYP4F11 is elevated in NASH HCCs, products of ω-hydroxylation are likely to increase and were mitochondrial enzymes and β-oxidation to be suppressed, transported to peroxisomes, undergo chain shortening and used for lipid biogenesis.

Another elevated cytochrome isozyme, CYP8B1 affecting the solubility of cholesterol and regulating the quantities of cholic and chenodeoxycholic acids, is regulated by SREBP-1c and localized in the endoplasmic reticulum membrane [18]. Previously, SREBP-1c has been reported to play an important role in development of IR and high-fat-induced obesity in the liver. Adaptive increase of fatty acid oxidation-related genes expression, such as PPARα, is induced by a high-fat diet. Nevertheless, a loss of ability to induce oxidation in response to high-fat diet is resulted in IR and obesity [19]. It is important to mention that HCV core has been shown be able to synthesize SREBP-1 but to activate PPARγ, which are suggested as mechanisms underlying steatosis development in HCV^+^ patients [20].

Nuclear receptors are considered extremely important for maintaining normal liver function. Recently, it was reported that several examples have roles in NAFLD and NASH development. Particularly important are members of the NR1 subfamily which are retained in the nucleus and form heterodimers with retinoid X receptors (RXRα, β, γ). These include PPARs (α, β, γ), liver X receptors (α, β), the farnesoid X receptor α (FXR), constitutive androstane receptor (CAR), and pregnane X receptor (PXR) [21]. All of these control carbohydrate and lipid homeostasis in gut:liver:adipose tissue and cross-talk between them has been proposed [21]. Our data support previously published results suggesting that hepatic nuclear receptors modulating genes controlling fatty acid and bile acid metabolism, including liver nuclear receptors PPARs, CAR, PXR and FXR, are inactivated in NASH patients [21,22,23,24].

It is known that PPARs are very important for suppression of NAFLD and NASH. PPARα regulates β-oxidation of fatty acids and elimination of cholesterol, while PPARγ is mostly present in adipose tissue and coordinates metabolism via transcription of adiponectin. PPARα is highly expressed with the liver ligand-activated nuclear receptor, which works as a nutritional sensor allowing adaptation regarding fatty acid catabolism, lipogenesis and synthesis of ketone bodies [25]. Its coactivators include different CBP/p300 and SRC/p160 family members. PPARα agonists are reported to act in association with LXR or insulin to induce lipogenesis and to enhance the activity of the human SREBP-1c promoter via a DR1 element at −453 [26]. On the other hand, insulin and cholesterol derivatives were shown to control SREBP-1c expression [26]. In support of previous data, in the present study, significant prediction of PPARγ suppression as well as decrease in protein expression of numerous mitochondrial enzymes involved in PPARα-controlled β-oxidation of fatty acids was found for NASH-associated HCCs. Thus, expression of human hydroxymethylglutaryl CoA synthase 2 (HMGCS2) was suppressed, which is known to be regulated by PPARα, but also interact with it and act as a co-activator [27]. HMGCS2 controls the 3-hydroxy-3-methylglutaryl-CoA (HMG-CoA) cycle, by which acetoacetate, β-hydroxybutyrate, and NAD^+^ are generated. In HepG2 cells, wild type human HMGCS2 expression induces both fatty acid oxidation and ketogenesis [27]. Here we detected 2.2-fold elevation of EHHADH, downstream protein of PPARα involved in fatty acid β-oxidation in peroxisomes, in NASH HCCs. In contrast, PPARα-controlled enzymes HMGCS2, ACCA2 and FABP1 were downregulated, pointing to inhibition of β-oxidation in mitochondria.

Treatment of rats and mice with PPARα and PPARγ agonists has been shown to markedly improve the histological picture of livers in NASH animals, with concurrently increased mRNA and protein expression of adiponectin receptors 1 and 2 in liver and visceral fat [28,29,30,31]. Furthermore, PPARγ has been shown to exert a suppressive effect on the invasive and metastatic potential of HCC cells in vitro and in vivo [32]. Recent data indicated that pharmacological activation of PPARα improves the metabolic milieu, hepatocyte ballooning and steatosis, and controls NFκB and JNK activation, neutrophil and F4/80 macrophage recruitment in diabetes-related NASH, but persistent liver inflammation with high serum MCP1 due to unsuppressed inflammation could limit PPARα agonist efficacy as a therapy for NASH [33]. PPARα has been further shown to be involved in protein folding and regulation of genes that protect the proteome [34]. An association of endoplasmic reticulum (ER) stress with NASH has also been previously reported [35].

To our knowledge, the activation of NRIP1 detected in human NASH biopsies is an interesting novel finding of the present study. Besides the modulatory activity of transcriptional activation by steroid receptors such as NR3C1, NR3C2 and ESR1, recently it has become clear that NRIP1 may affect oxidative metabolism and mitochondrial biogenesis by negatively controlling mitochondrial pathways regulated by PPARγ coactivator 1 α (PGC-1α). Furthermore, the NRIP1-PGC-1α axis might represent a potential therapeutic target for restoring altered mitochondrial function in the Down’s syndrome [36]. Thus, it could be speculated that mitochondrial dysfunction in NASH might be restored when targeting the NRIP1-PGC-1α pathway. NRIP1 knockout mice are lean and stay lean, even on a rich diet. The NRIP1gene has further been reported to be implicated in the control of energy expenditure, behavior, cognition, mammary gland development, intestinal homeostatic regulation of various oncogenic signaling pathways and development and progression of solid tumors and hematologic malignancies such as chronic lymphocytic leukemia [37]. From our results, NRIP could become the new useful target in NASH.

Cells are usually damaged by the high ROS levels, however, ROS could also protect from tumorigenesis serving as a barrier [38]. Therefore, activation of defense mechanisms in NASH pathogenesis could be a result of the persistent high levels of inflammation and ROS in the liver [38]. In the progression stage, cancer cells are often exposed to high levels of ROS, and their ability to escape the consequences of this is extremely important for cancer cells to survive and propagate. It has been suggested, that tumor cells could become resistant to oxidative stress, due to the activation of Nrf2 [39]. In our study, in both types of HCCs, NASH-related and HCV^+^, two downstream proteins of Nrf2, namely, NQO1 and SOD2, which participate in ROS elimination, were found induced, indicating that Nrf2 plays an important role in adaptation to oxidative stress in the tumor. Its recognizes a unique DNA sequence known as the antioxidant response element (ARE), and positively regulates the expression of a battery of genes participating in protection against oxidative/electrophilic stress, with possible roles in mediating interrelations between lipid metabolism and antioxidant defense mechanisms in experimental models of NAFLD [39,40]. Our data are in line with previously published results indicating stabilization of Nrf2 and upregulation of Nrf2 activity in NASH [41,42].

Elevation of expression of proteins induced in response to formation of oxidative stress in NASH and HCV^+^ HCCs was clearly seen from our proteome analysis results. Thus, expression of mitochondrial prohibitins, which is usually elevated in response to oxidative stress, was detected. Prohibitins inhibit DNA synthesis, contribute to control of mitochondria respiration and function, and negatively regulate cell proliferation, while acting as repressors of transcription via recruitment of histone deacetylases. Furthermore, they may be induced in response to IL6 and suppress apoptosis and appear important for generation of the TGF-β-mediated mesenchymal cell phenotype [11,12].

In the present study β-catenin band wnt1 staining was observed in the cytoplasm and/or nuclei of tumor cells as well as in oval cells within HCCs, indicating activation of Wnt1 signaling in human NASH-associated and STAM mice hepatocarcinogenesis. Wnt proteins are highly conserved across species and comprise a diverse family of signaling glycoproteins 350–400 amino acids in length featuring lipid modification (palmitoylation of cysteines) [43,44]. It has been reported that palmitoylation and glycosylation of Wnt proteins are necessary to initiate their targeting to the plasma membrane for secretion and binding with receptors due to the covalent attachment of fatty acids [45] resulting in activation of different Wnt pathways via paracrine and autocrine routes [46,47]. Proliferation and growth of embryonic stem cells and tissue homeostasis has been reported to be mediated through canonical Wnt/β-catenin (Wnt) signaling, which increases nuclear and cytoplasmic β-catenin, acting as a transcriptional coactivator of transcription factors from the TCF/LEF family [48,49]. Its activation occurs if a Wnt ligand binds to a Frizzled (FZD) receptor at the cell membrane. Without Wnt signaling, β-catenin can not accumulate in the cytoplasm since a destruction complex would normally degrade it [49]. In general, the undifferentiated state of stem cells is maintained by Wnt proteins, while other growth factors, such as FGF and EGF, participating in signaling through tyrosine kinase pathways, force cells to proliferate [50]. Aggressive HCC phenotype characterized by increased cell proliferation, survival, migration and invasion is associated with deregulation of the Wnt pathway, which is an early event in hepatocarcinogenesis [49,51]. It has been suggested that Wnt signaling is involved in the key process of epithelial–mesenchymal transition (EMT), particularly during mammary development [52]. In the process of EMT, which involves cadherin down-regulation, epithelial cells are transformed into mesenchymal cells, thus becoming no longer held in place by laminin. In addition, in this study we observed activation of Stat3, known to be responsive to leptin through the Janus kinase (Jak) 2 signal transducer. Leptin has been reported to protect against hepatic steatosis, increasing β-catenin levels which might contribute to leptin regulation of SREBP-1c expression in hepatic stellate cells (HSCs) [53]. Hepatocytes and cholangiocytes are differentiated from liver oval cells, which are the highly proliferative postnatal progenitors with bipotent differentiation ability [54]. They respond with an increase in amino-terminus dephosphorylated β-catenin and cell-cycle entry to the Wnt ligands (Wnt3a) in vitro [54]. Furthermore, in vivo studies reported, that Wnt/β-catenin/TCF pathway is activated in proliferating facultative hepatic progenitor cells [54].

Increased Wnt signals could act as a potent activator of mitochondrial biogenesis and ROS generation, leading to DNA damage and acceleration of cellular senescence. Furthermore, Wnt proteins have been suggested to be able to modulate glucose homeostasis through insulin receptor substrate-1 (IRS-1) which drives activation of mitochondrial biogenesis and enhances insulin signaling in insulin-responsive cell types [55]. Transcription factor TCF7L2, involved in Wnt signaling, has been reported to play an important role and to be one of the risk factors in type 2 diabetes [56,57]. In this context, it is interesting that ROS may play an important role in the development of acute hepatic IR and activation of stress signaling after injury [58].

In human NASH-associated HCCs suppression of CK8/18 production was here indicated by proteome and immunohistochemical results. This could be a result of CK8/18 fragmentation and degradation in NASH HCCs, as proposed in previous studies. Interestingly, we observed contrasting increase in STAM mice HCCs. CK8 and CK18 were previously shown to be upregulated in livers of HCV^+^ patients with more active disease, but not in HBV/NASH subjects [59].

The increase of CK8/18 in mouse HCCs was accompanied by significant (3.6-fold) up-regulation of glutathione S-transferase Mu 1 (GSTM1), an enzyme involved in gluthathione metabolism. Overexpression of PHB1 and PHB2 and development of mitochondrial stress was found in both NASH-associated human and STAM mice HCCs. However, induction of PPARα-inducible EHHADH, an enzyme controlling the peroxisomal β-oxidation of fatty acids, was detected only in STAM mice. Thus, in HCCs of these animals, fatty acid β-oxidation is likely to be activated in peroxisomes.

## 4. Experimental Section

### 4.1. Chemicals

All reagents were purchased from Wako Pure Chemicals Industries (Osaka, Japan) or Sigma (St. Louis, MO, USA).

### 4.2. Approval of the Institutional Review Board and Informed Consent

This study was approved by the Ethics Committee (No. 2263; 14AS, 25 July 2014) of Osaka City University Graduate School of Medicine (Osaka, Japan). It was performed according to the principles of the Declaration of Helsinki. Informed consent was obtained from all patients prior to the study.

### 4.3. Patients and Tissue Specimens

This prospective study included liver biopsies at high fibrosis stages 3 and 4 and primary HCCs obtained from 10 NASH patients each, HCCs from 10 HCV^+^ patients and normal-appearing liver tissue specimens from 10 patients with metastasis from gastrointestinal cancers, undergoing biopsy or operations at Osaka City University Hospital (Osaka, Japan) from January 1998 to December 2011. A complete histopathological analysis was performed for all specimens to prove NASH, HCC or metastatic lesions.

Clinicopathological characteristics of the patients are presented in Supplementary Tables S1 and S2. Two male and 8 female patients diagnosed NASH (median age 62 years (range 47–71 years), 8 male and 2 female NASH patients with primary HCC (median age 71 years (range, 54–79 years)), 6 male and 4 female HCV^+^ patients with primary HCC (median age 70 years (range, 45–84 years)) and 7 male and 3 female patients with colon and colorectal cancer (adenocarcinoma) metastasis to the liver (median age 63 years (range, 47–77 years)) at the time of biopsy or surgery, were enrolled in the study. Each biopsy was evaluated using the NAFLD Activity Score (NAS) and Brunt’s criteria. NAS was counted as the sum of the separate scores for steatosis (0–3), hepatocellular ballooning (0–2) and lobular inflammation (0–3). For the Brunt’s criteria, separate assessments of steatosis (grades 0–3), necroinflammatory lesions (grades 1–3) and fibrosis (stages 1–4) were used. The Japanese classification of HCCs was used for histopathological analysis and staging [60]. The general guidelines for primary liver cancer of the American Joint Committee on Cancer/International Union Against Cancer staging systems [61] and the Liver Cancer Study Group of Japan [60] were taken as the basis for the pathological diagnoses which were performed by at least two pathologists in our hospital. Six of 10 NASH HCC and 4 of 10 HCV^+^ HCC patients were diagnosed with recurrence.

### 4.4. QSTAR Elite LC-Ms/Ms

To perform the proteome analyses, 10% phosphate-buffered formalin-fixed paraffin embedded (FFPE) biopsy and resected HCC samples were applied. For every case histopathological analysis was carried out prior to microdissection or needle dissection. Dissected samples were then dissolved in a solution optimized for the proteome analysis of FFPE sections (Liquid tissue lysis buffer, AMR, Tokyo, Japan), heated to 90 °C for 90 min and digested with trypsin at 37 °C for 16~18 h. Analysis of proteome signatures in the tissue samples (20 µg each) was performed with Da iNa-AI nano Liquid Chromatograph (LC) (KYA Technologies, Tokyo, Japan) coupled to a QSTAR Elite hybrid mass spectrometer (AB Sciex, Concord, ON, Canada) with a NanoSpray ion source (AB Sciex, Concord, ON, Canada) as previously described [62]. For quantitative assessment, peptide labeling with 4-plex iTRAQ reagents was employed [62]. The samples were labeled as follows 114, NASH biopsies; 115, NASH HCCs; 116, HCV^+^ HCCs; and 117, pooled control samples of normal-appearing liver tissues of gastrointestinal tumor metastasis patients. Protein identification was performed from Ms/Ms data using ProteinPilot software 2.0 (AB Sciex, Tokyo, Japan) with the Paragon Algorithm for redundant hits removal and comparative quantitation, resulting in the minimal set of justifiable identified proteins, by comparison against the Swiss Protein database (HUMAN) with trypsin set as the digestion enzyme and methyl methanethiosulfonate for cysteine modification. Each sample was run for 150 min and measured 3 times and all reported data were used at 95% confidence cut-off limits created by ProteinPilot software. The same program was applied to remove the bias for proteins expressed at very low levels. Protein levels were quantified by comparing iTRAQ reporter ion intensities among NASH biopsies, HCCs, HCV^+^ HCCs and the normal-appearing tissue samples with a *p*-value cut-off of < 0.05 (Table S7). The averaged LC-Ms/Ms and ProteinPilot analysis results from 10 patients with protein expression alterations observed in more than 50% of cases were uploaded to perform Ingenuity Pathway Analysis (IPA) (Ingenuity Systems, Mountain View, CA, USA) aimed to investigate altered molecular functions, protein localization, networks and signaling pathways, as well as activated or suppressed upstream regulators associated with NASH and to perform the comparative analysis of NASH-associated and HCV^+^ HCCs.

### 4.5. Ingenuity Pathway Analysis (IPA)

The Ingenuity program (Ingenuity Systems, Mountain View, CA, USA) was used to assign biological significance to differentially labeled proteins and further to identify activated or suppressed upstream regulators, altered functional groups and pathways proteins known to be associated with cancer biology were prioritized for further validation.

### 4.6. STAM Mice, Experimental Design and Histopathology

Seven male 6-week-old male STAM mice (C57BL/6N-NASH) were obtained from Charles River laboratories Inc. (Kanagawa, Japan). In the present NASH model (experiment approval No. AE09-034), C57BL/6N strain mice were injected with streptozotocin at 2 days of age and 4 weeks later administered high fat diet (D12492), resulting in induction of insulin resistance without genetic change. Six weeks after the streptozotocin injection animals were purchased and given free access to tap water and high-fat diet (HFD-60, Oriental Yeast Co., Tokyo, Japan). At 17 weeks of age euthanization was performed to allow macroscopic, microscopic and proteome analysis, with histopathological and immunohistochemical assessment.

### 4.7. Proteome Analysis in STAM Mice HCCs

Formalin-fixed tumors developing in STAM mice histologically diagnosed as HCCs were subjected to quantitative proteome analysis using iTRAQ labeling and QSTAR-Elite Ms/Ms by the method described previously [62]. Pooled samples from microdissected hematoxylin-stained formalin-fixed and paraffin embedded (FFPE) sections of HCCs from STAM mice and normal-appearing livers from five non-treated 17 week-old C57BL/6N male mice (Charles River Laboratories Japan Inc., Kanagawa, Japan) were subjected to analysis. Because of the difficulties in collection of STAM mouse HCCs tissue by microdissection, it was decided to use pooled samples for proteome analysis. The liquid tissue lysis buffer was chosen and adjusted specifically for use with FFPE sections. Previously, comparative analysis of frozen tissue and FFPE sections LC-Ms/Ms of different animal tissues showed high concordance of results. Duplicate pooled samples from microdissected and/or needle dissected 10% phosphate-buffered formalin-fixed mouse HCCs and normal-appearing liver tissue of C57Bl/6N mice were prepared and labeled as follows: iTRAQ isobaric reagents 114, STAM mouse HCCs; 115, normal-appearing liver from control C57Bl/6N mice. Swiss Protein database (MOUSE) and ProteinPilot™ software (version 2.0, AB Sciex, Concord, ON, Canada) were employed for the analysis of Ms/Ms data with trypsin as the digestion enzyme and methyl methanethiosulfonate for cysteine modification. The protein ratios were considered significant at *p*-value less than 0.05.

### 4.8. Immunohistochemical Examination

Single immunohistochemistry for β-catenin, wnt1, CK8, CK18, PHB1 and PHB2 was performed for human NASH-associated and HCV^+^ HCCs and STAM mice HCCs using the ABC method as previously reported [63]. A solution of 3,3′-diaminobenzidine tetrahydrochloride (DAB) (DAKO, Tokyo, Japan) was applied for visualization of target proteins. Paraffin sections from normal livers of C57Bl/6N mice were used for comparison. We generated new rat monoclonal CK8 CK18, PHB1 and PHB2 antibodies using the rat lymph node method (all from Risk Assessment and Research Inc., Japan) [64]. Rabbit polyclonal antibodies against β-catenin (dilution 1:100; Abcam Co., Tokyo, Japan) and wnt 1 (dilution 1:80; Abcam Co., Japan) were employed for immunohistochemical evaluation of protein expression and activation of the wnt signaling pathway in human and STAM mice HCCs. Immunohistochemical procedures were optimized by testing different antigen retrieval methods and negative controls.

### 4.9. Statistical Analysis

The significance of differences between mean values was analyzed by the two-tailed Student *t*-test using the StatLight–2000(C) program (Yukms Corp., Tokyo, Japan). For analysis of protein expression, statistical analysis with ProteinPilot^TM^ 2.0 Software was employed. We have performed the clustering analysis of our samples using the IPA. Furthermore, we performed the principal component analysis and statistically evaluated associations between expression of proteins, and activation scores of upstream regulators with clinicopathological variables using *χ*-square and Fisher’s tests using the SPSS for the most highly distinct characteristics.

## 5. Conclusions

In the present study, activation of IGF1 and TGF-β was associated with up-regulated Wnt/β-catenin signaling, NRIP1, Nrf2 signaling and decrease of CK8 and CK18 levels in human NASH HCCs, correlated with suppression of PPARs, HNF4A, CAR, PXR and, in contrast, overexpression of PHBs and activation of SREBP-1 and LXRα, leading to enhanced lipid biogenesis and cholesterol synthesis, development of oxidative stress and suppression of mitochondrial function. In STAM NASH mice livers, PPARs inhibition was not evident and CK8 and CK18 levels were high, indicating the existence of major differences in human and mouse mechanisms of NASH pathogenesis.

## Figures and Tables

**Table 1:** Differentially expressed proteins in NASH-associated biopsies and HCCs, and HCV^+^ HCCs, identified by QSTAR Elite LC-Ms/Ms.

Protein Name (Symbol)	GI Number	NASH Biopsy		NASH HCC		HCV^+^ HCC		Location	Function(s)
		Ratio	*p*-Value	Ratio	*p*-Value	Ratio	*p*-Value		
Collagen α-1(I) chain (COL1A1)	124056487	1.52	0.000	1.24	0.000	1.27	0.000	ES	EMO
Collagen α-1(II) chain (COL2A1)	124056489	1.20	0.049	1.29	0.011	1.39	0.026	ES	EMO
Collagen α-1(III) chain (COL3A1)	124056490	1.75	0.000	1.23	0.000	0.98	0.098	ES	EMO
Collagen α-1(VI) chain (COL6A1)	125987811	1.20	0.002	1.26	0.047	1.10	0.195	ES	EMO
Collagen α-2(I) chain (COL1A2)	124056488	1.56	0.000	1.27	0.000	1.71	0.000	ES	EMO
Collagen α-2(IV) chain (COL4A2)	143811377	1.23	0.012	1.22	0.043	1.11	0.212	ES	EMO
Collagen α-2(VI) chain (COL6A2)	125987812	1.32	0.000	1.24	0.000	0.84	0.307	ES	EMO
Collagen α-3(VI) chain (COL6A3)	215274244	1.21	0.000	1.25	0.012	0.95	0.298	ES	EMO
Fibronectin (FN)	2506872	1.23	0.004	2.06	0.000	1.94	0.000	ES	EMO
Lumican (LUM)	20141464	1.51	0.037	1.22	0.012	1.25	0.430	ES	EMO
Biglycan (BGN)	266762	1.31	0.003	1.36	0.002	0.54	0.503	ES	EMO
Putative annexin A2-like protein (ANXA2P2)	205830271	1.26	0.018	1.40	0.012	0.96	0.577	ES	EMO,CaPhB
Vimentin (VIM)	55977767	1.53	0.000	1.41	0.000	1.22	0.000	C	CO,EMT
β-actin-like protein 2 (ACTBL2)	172046825	1.25	0.000	1.24	0.000	1.20	0.000	C,N	CO
Myosin-9 (MYH9)	6166599	1.20	0.000	1.25	0.000	1.22	0.000	C	CO
Tropomyosin α-4 chain (TPM4)	54039746	1.41	0.000	1.30	0.000	0.84	0.007	C	CO
Tubulin α-1C chain (TUBA1C)	20455322	1.20	0.000	1.45	0.000	1.42	0.001	C	CO
Keratin, type II cytoskeletal 8 (KRT8, CK8)	90110027	0.73	0.000	0.77	0.000	1.24	0.000	C	CO
Keratin, type I cytoskeletal 18 (KRT18, CK18)	125083	0.75	0.000	0.80	0.000	1.42	0.049	C	CO
Lymphocyte cytosolic protein 1 (LCP1)	1346733	1.21	0.046	1.45	0.025	1.01	0.873	C	CO,ACO
Moesin (MSN)	127234	1.24	0.018	1.28	0.000	0.96	0.529	PM	CO,CA
Superoxide dismutase [Mn], mitochondrial (SOD2)	134665	0.82	0.000	2.13	0.000	0.97	0.827	C,M	ORP
Catalase (CAT)	115702	0.95	0.138	0.74	0.000	0.90	0.105	C	ORP,CTM,AM
Thioredoxin (TXN)	135773	0.98	0.56	1.28	0.005	0.95	0.113	C,N	ORP,PF,ST
Glutathione S-transferase κ1 (GSTK1)	12643338	0.98	0.726	1.22	0.008	1.01	0.988	C,M	GM
Glutathione S-transferase A1 (GSTA1)	121730	0.72	0.000	0.45	0.000	0.968	0.943	C,EE	XM,GM
Epoxide hydrolase 1 (EPHX1)	123926	0.89	0.000	0.81	0.000	1.16	0.441	C,EPR	XM
UDP-glucuronosyltransferase 2B17 (UGT2B17)	6136104	0.99	0.928	0.74	0.001	1.17	0.158	C,EPR	ORP,PF
UDP-glucuronosyltransferase 2B7 (UGT2B7)	136727	0.95	0.327	0.58	0.000	0.96	0.42	C,EPR	XM
Protein disulfide-isomerase A4 (PDIA4)	119530	0.96	0.211	1.22	0.007	1.31	0.000	C,EPR	ORP,XM
Protein disulfide-isomerase (P4HB)	2507460	0.96	0.036	1.27	0.000	1.29	0.000	C,EPR	ORP,PF
Cytochrome P450, fam.2, subfam. C, polypep.8 (CYP2C8)	117225	0.64	0.000	0.64	0.000	0.94	0.761	C,EPR	ORP,XM
Cytochrome P450, fam.2, subfam. C, polypep.9 (CYP2C9)	6686268	0.94	0.157	0.75	0.000	1.14	0.097	C,EPR	ORP,XM
Cytochrome P450, fam.4, subfam. F, polypep.11 (CYP4F11)	20532035	1.03	0.52	1.89	0.000	0.91	0.185	C,EPR	ORP,SREBP,FAOH
Cytochrome P450, fam.51, subfam. A, polypep.1 (CYP51A1)	3915660	0.95	0.398	2.13	0.000	1.21	0.452	C,EPR	LCB,SREBP,LXRα
Cytochrome P450, fam.8, subfam. B, polypep.1 (CYP8B1)	13124098	0.91	0.179	2.00	0.000	0.61	0.020	C,EPR	BAB,SM,SREBP1c
Enoyl-CoA hydratase, mitochondrial (ECHS1)	62906863	0.87	0.000	0.60	0.000	1.01	0.951	C,M	FABO
3-ketoacyl-CoA thiolase, mitochondrial (ACAA2)	57015371	0.87	0.000	0.60	0.000	1.23	0.244	C,M	FAM,FABO,PPARS
Fatty acid-binding protein, liver (FABP1)	119808	0.76	0.000	0.57	0.000	1.08	0.673	C	FABO,AA,PPARS
Peroxiredoxin-6 (PRDX6)	1718024	0.80	0.000	0.79	0.000	2.63	0.000	C	LCP,ORP,PPARS
Apoptosis-inducing factor 1, mitochondrial (AIFM1)	13431764	0.82	0.027	0.55	0.002	0.78	0.002	C,M,N	A(+),ORP
Cell death regulator Aven (AVEN)	20454834	1.12	0.045	1.32	0.034	0.98	0.784	EMS,N	A(−)
Annexin A6 (ANXA6)	113962	1.01	0.759	0.86	0.000	0.93	0.117	PM	A(+),Ca PhB
Alcohol dehydrogenase 1B (ADH1B)	113394	0.77	0.000	0.44	0.000	0.86	0.161	C	AM,EO
Alcohol dehydrogenase 1C (ADH1C)	113398	0.81	0.000	0.56	0.000	0.94	0.262	C	AM,EO
Alcohol dehydrogenase 4 (ADH4)	83286923	0.83	0.000	0.42	0.000	0.93	0.854	C	AM,EO
Aldehyde dehydrogenase, mitochondrial (ALDH2)	118504	0.80	0.000	0.57	0.000	1.25	0.022	C,M	AM
Prohibitin 1, mitochondrial (PHB1)	4505773	0.96	0.679	1.81	0.001	1.25	0.008	C,M,N	MF,T
Prohibitin 2, mitochondrial (PHB2)	76363296	1.60	0.037	2.99	0.000	1.54	0.005	C,M,N	MF,T
Cytochrome b-c1 complex subunit 2, mitochondrial (MT-CYB)	21903482	0.95	0.186	0.74	0.000	1.15	0.324	C,M	MT
Aldehyde dehydrogenase family 4 member A1 (ALDH4A1)	62511241	0.99	0.917	0.77	0.000	1.08	0.196	C,M	ProM
Glutamate dehydrogenase 1, mitochondrial (GLUD1)	118541	0.92	0.000	0.69	0.000	1.09	0.991	C,M	GluM,PPARS
Aspartate aminotransferase, mitochondrial (GOT2)	112983	0.93	0.005	0.71	0.000	0.96	0.919	C,M	AsM,FAT,PPARS
Ornithine carbamoyltransferase, mitochondrial (OTC)	84028235	1.07	0.054	0.72	0.000	1.14	0.340	C,M	UC,PPARS
Carbamoyl-phosphate synthase [ammonia], mitochondrial (CPS1)	4033707	0.78	0.000	0.46	0.000	1.05	0.769	C,M,N	UC,PPARS
Arginase-1 (ARG1)	12230985	0.92	0.015	0.80	0.000	1.501	0.091	C,M	UC,PPARS
Acetyl-CoA acetyltransferase, mitochondrial (ACAT1)	135755	0.91	0.023	0.67	0.000	1.25	0.000	C,M	KM,ATD,PPARS
Hydroxymethylglutaryl-CoA synthase, mitochondrial (HMGCS2)	1708234	0.78	0.000	0.64	0.000	1.10	0.098	C,M	KM,ATD,PPARS
4-aminobutyrate aminotransferase, mitochondrial (ABAT)	48429239	0.79	0.000	0.56	0.000	0.92	0.498	C,M	GABAM
Heat shock 70 kDa protein 1 (HSP70)	75061728	1.08	0.059	1.36	0.000	1.91	0.000	C	PF
Heat shock protein HSP 90-α (HSP90AA1)	92090606	1.02	0.723	1.22	0.000	1.69	0.000	C,N	PF,SR
Heat shock protein HSP 90-β (HSP90AB1)	17865718	1.01	0.702	1.77	0.000	1.47	0.000	C,ES	PF,SR
78 kDa glucose-regulated protein (GRP-78; HSPA5)	14916999	0.99	0.689	1.45	0.000	1.35	0.000	C,EPR	PF,PRCM
Histone H2A type 1 (H2A1)	84028211	1.37	0.000	1.40	0.000	1.66	0.000	N	ChS
Histone H3.1t (H31T)	18202512	1.29	0.000	1.36	0.000	1.47	0.000	N	NA

C: cytoplasm; CS, cytoskeleton; EE, extracellular exosome; EMS, endomembrane system; EPR, endoplasmic reticulum; ES: extracellular space; L, lysosome; M, mitochondria; N: nucleus; PM: plasma membrane. A(+) and A(−), positive and negative regulation of apoptosis, respectively; AA, antioxidant activity; ACO, actin cytoskeleton organization; AM, alcohol metabolism; AsM, aspartate metabolism; ATD, adipose tissue development; BAB, bile acid biosynthesis; CA, cell adhesion; CaPhB: Ca-dependent phospholipid binding; ChS, chromatin silencing; CO, cytoskeleton organization; CTM, cholesterol-triglyceride metabolism; EMO, extracellular matrix organization; EMT: epithelial mesenchymal transition; EO, ethanol oxidation; FAOH, fatty acid omega hydroxylation; FABO, FAM and FAT fatty acid β oxidation, metabolism and transport, respectively; GABAM, GABA metabolism; GM, glutathione metabolism; GluM, glutamate metabolism; LCB, lipid and cholesterol biosynthesis; KM, ketone body metabolism; LCP, lipid catabolic process, MF, mitochondrial function; MT, mitochondrial transport; NA, nucleosome assembly; ORP, oxidation-reduction process; PF, protein folding; PPARS, PPAR signaling; PRCM, positive regulation of cell migration; ProM, proline metabolism; SM, sterol metabolism; SR, stress response; SREBP, sterol regulatory element-binding proteins; ST, signal transduction; T, transcription; UC, urea cycle; XM, xenobiotic metabolism; “0.000, *p* < 0.0001”.

**Table 2:** Differentially expressed proteins in the HCCs of STAM mice identified by QSTAR Elite LC-Ms/Ms.

Name	GI Number	Ratio	*p*-Value	Location	Functions
Glutathione S-transferase Mu 1 (GSTM1)	6754084	3.63	0.000	C	GM
Keratin, type II cytoskeletal 8 (KRT8, CK8)	114145561	1.40	0.0093	C,N	CO,CA,AP
Keratin, type I cytoskeletal 18 (KRT18, CK18)	254540068	1.33	0.0019	C	CO,CA,AP
Actin, cytoplasmic 2 (ACTG1)	6752954	1.25	0.0005	C,CS	CO,CA
Peroxiredoxin-1 (PRDX1)	6754976	1.34	0.0084	C	ORP,CP,RSS
Peroxisomal bifunctional enzyme (EHHADH)	31541815	2.16	0.000	P	LM,FABO,PPARS
Thioredoxin-dependent peroxide reductase, mitochondrial (PRDX3)	6680690	2.36	0.013	M	ORP,CP,NRA,NFκBPR
Annexin 5 (ANXA5)	6753060	1.68	0.025	C	PLB,NRA
Prohibitin 1 (PHB1, PHB)	6679299	2.25	0.000	M,C,N	TR,MF
Prohibitin 2 (PHB2)	126723336	2.76	0.000	M,C,N	TR,MF
Hydroxymethylglutaryl-CoA synthase, mitochondrial (HMGCS2)	31560689	0.87	0.016	M	ATD,MB,CRI,FABO,PPAR
3-ketoacyl-CoA thiolase, mitochondrial (ACCA2)	29126205	0.80	0.001	M	LM,FAM,AP,PPARS
Fatty acid-binding protein, liver (FABP1)	8393343	0.89	0.0438	C	LM,CUH,FAB,AOA,PPARS
Argininosuccinate synthase (ASS1)	6996911	0.53	0.000	C,M,EPR	AAM,AB,APR
ATP synthase subunit α, mitochondrial (ATP5A1)	6680748	0.84	0.0429	M	EM,ATPB
60 kDa heat shock protein, mitochondrial (HSPD1)	183396771	0.78	0.0445	M	PF
Major urinary protein 2-like precursor (MUP2l)	530354677	0.24	0.041	C	IARA,CRL,PRGM
Betaine-homocysteine S-methyltransferase 1 (BHMT)	7709990	0.39	0.000	C	AAM,CC,LM
Fructose-bisphosphate aldolase B (ALDOB)	21450291	0.76	0.0014	C,CS,MT	CM,GP,CRI
Glutamate dehydrogenase 1, mitochondrial (GLUD1)	6680027	1.30	0.0433	M	TCA,AAM
Malate dehydrogenase, mitochondrial (MDH2)	31982186	1.59	0.0399	M	CM,MM
Ornithine aminotransferase, mitochondrial (OAT)	8393866	2.47	0.000	M	AAM,AC,PB
Hemoglobin, β adult t chain (HBB)	319823001	1.48	0.02	C,ES	OT,ORP,PRCD

C, cytoplasm; CS, cytoskeleton; EPR, endoplasmic reticulum; ES, extracellular space; M, mitochondria; MT, microtubule; N, nucleus; P, peroxisome. AAM, amino acid metabolism; AB, arginine biosynthesis; AC, arginine catabolism; AP, apoptotic process; APR, acute phase response; AOA, antioxidant activity; ATD, adipose tissue development; ATPB, ATP biosynthesis; CA, cell-cell adhesion; CC, choline catabolism; CM, carbohydrate metabolism; CO, cytoskeleton organization; CP, cell proliferation; CRI, cellular response to insulin; CRL, cellular response to lipid; CUH, cholesterol uptake in hepatocytes; EM, energy metabolism; EO, ethanol oxidation; FAB, fatty acid binding; FABO, fatty acid β-oxidation; GM, glutathione metabolism; GP, glycolytic process; IARA, insulin activated receptor activity; LM, lipid metabolism; MB, mevalonate biosynthesis; MF, mitochondria function; MM, malate metabolism; NFκBPR, NFκB positive regulation; NRA, negative regulation of apoptosis; ORP, oxidation-reduction process; OT, oxygen transport; PF, protein folding; PB, proline biosynthesis; PLB, phospholipid binding; PPARA, PPARα; PRGM, positive regulation of glucose metabolism; PRCD, positive regulation of cell death; PRLM, positive regulation of lipid metabolism; RSS, removal of superoxide radicals; TCA, tricarboxylic acid metabolism, TR, transcription.

## Supplementary Materials

Supplementary materials can be found at www.mdpi.com/1422-0067/18/2/434/s1.
