# Supplementary material for: Proteome Characteristics of Non-Alcoholic Steatohepatitis Liver Tissue and Associated Hepatocellular Carcinomas
Source: Int J Mol Sci. 2017 Feb 17;18(2):434. doi: 10.3390/ijms18020434 (PMC5343968; doi:10.3390/ijms18020434)
Supplement: Supplementary file 1 [file ijms-18-00434-s001.zip › ijms-169550 SI.pdf]

Supplementary Materials: Proteome Characteristics of Non-Alcoholic Steatohepatitis Liver Tissue and Associated Hepatocellular Carcinomas

Anna Kakehashi, Vasily E. Stefanov, Naomi Ishii, Takahiro Okuno, Hideki Fujii, Kazuaki Kawai, Norifumi Kawada and Hideki Wanibuchi

Table S1. Clinicopathological features, tumor and treatment characteristics for NASH-associated and HCV⁺ HCC cancer patients.

		NASH HCC			HCV⁺ HCC			Metastatic tum. Normal Liver		
		**Median (Range)**	***N***	**%**	**Median (Range)**	***N***	**%**	**Median (Range)**	***N***	**%**
	**Patient demographics**									
Gender	Male		8	80		6	60		7	70
	Female		2	20		4	40		3	30
Cirrhosis stage	1		2	20		1	10		0	0
	2		6	60		5	50		1	10
	3		1	10		1	10		0	0
	4		1	10		3	30		0	0
Age (y)	>70	75.2 (71–79)	4	40	75.2 (71–84)	4	40	74.0 (71–77)	2	20
	≤70	63.8 (54–69)	6	60	61.0 (45–67)	6	60	59.9(47–69)	8	80
BMI	15–24	0 (0)	0	0	21.3 (15.7–24.0)	8	80	22.1 (18.4–24.0)	7	70
	>25	28.1 (25.6–31.0)	10	100	27.4 (25.9–28.8)	2	20	25.9 (25.1–26.4)	3	30
Diabetes	Positive		7	70		1	10		2	20
	Negative		3	30		9	90		8	80
Alcohol	Drinker		0	0		3	30		8	80
	Non-drinker		10	100		7	70		2	20
Smoking	Smoker		7	70		8	80		4	40
	Non-smoker		3	30		2	20		6	60
AST	13–33 IU/L;		6	60		4	40		5	50
	>34 IU/L		4	40		6	60		5	50
ALT	6–27 IU/L		6	60		3	30		3	30
	>28 IU/L		4	40		7	70		7	70
ALP	115–359 IU/L		7	70		9	90		6	60
	>360 IU/L		3	30		1	10		4	40
	**Tumor characteristics**									
Tumor size (cm³)	≤3	2.4 (0.59−2.73)	3	30	0.9 (0.14−2.25)	6	60	0.4 (0.13−0.97)	6	60
	>3	100.4 (3.38- 367.5)	7	70	46.6 (3.00- 10.00)	4	40	46.6 (11.39- 101.25)	4	40
Depth of tumor invasion	T1		1	10		4	40		0	0
	T2		3	30		2	20		3	30
	T3		4	40		3	30		3	30
	T4		2	20		1	10		4	40
Differentiation	Well		0	0		0	0		0	0
	Moderate		10	100		10	100		7	70
	Poor		0	0		0	0		3	30
Clinical stage	I		1	10		4	40		0	0
	II		3	30		2	20		3	30
	III		4	40		3	30		3	30
	IV		2	20		1	10		4	40
Venous invasion	Positive Negative		4	40		2	20		-	-
			6	60		8	80		-	-
Recurrence	Positive		6	60		4	40		5	50
	Negative		4	40		6	60		5	50
Distant metastasis, pM	Positive		0	0		1	10		6	60
	Negative		10	100		9	90		4	40
Bile duct invasion, pB	Positive		1	10		0	0		-	-
	Negative		9	90		10	100		-	-
Formation of capsule	Present		7	70		7	70		-	-
	Absent		3	30		3	30		-	-
Infiltration to capsule	Positive Negative		6	60		6	60		-	-
			4	40		4	40		-	-

Table S1. *Cont.*

		NASH HCC			HCV+ HCC			Metastatic tum. Normal Liver		
		Median (Range)	*N*	%	Median (Range)	*N*	%	Median (Range)	*N*	%
**Treatment characteristics**										
Neoadjuvant therapy	Yes		3	30		2	20		9	90
	No		7	70		8	80		1	10
Radiation therapy	Yes		3	30		2	20		2	20
	No		7	70		8	80		8	80
Surgical resection	Lobe		2	20		0	0		4	40
	Wedge		8	80		10	100		6	60

Tumor volume was counted as length/2 * (width/2) * (width/2) and expressed in cm^3; B, Brunt Criteria.

Table S2. Clinicopathological characteristics of NASH biopsy patients.

		NASH Biopsy		
		Median (Range)	*N*	%
**Patient demographics**				
Gender	Male		2	20
	Female		8	80
Age (y)	>70	71 (71–71)	2	20
	≤70	59.6 (47–68)	8	80
BMI	15–24	0	1	10
	>25	28.7 (25.0–35.6)	9	90
Diabetes	Positive		6	60
	Negative		4	40
Alcohol	Drinker		0	0
	Non-drinker		10	100
AST	13–33 IU/L ;	0	0	0
	>34 IU/L	57.7 (34–74)	10	100
ALT	6–27 IU/L	0	0	0
	>28 IU/L	54.2 (31–93)	10	100
ALP	115–359 IU/L	226.1 (169–331)	9	90
	>360 IU/L	257 (169–472)	1	10
GGT		88.9 (48–256)	10	100
Alb		3.6 (3.0–4.2)	10	100
T-bil		1.3 (0.6–1.8)	10	100
D-bil		0.4 (0.2–0.8)	10	100
TG		130 (60–217)	10	100
Accumulation of lipids in hepatocytes	grade 1		6	60
	grade 2		4	40
Inflammation	grade 2<br>grade 3		4	100
Hepatocytes balooning	grade 1		1	10
	grade 2		9	90
NAS	grade 3		1	10
	grade 4		0	0
	grade 5		4	40
	grade 6		2	20
	grade 7		3	30
Accumulation of lipids in hepatocytes (B)	grade 1		3	30
	grade 2		5	50
	grade 3		2	20
Inflammation (B)	grade 2		5	50
	grade 3		5	50
Fibrosis (B)	grade 4		10	100
Matteoni	grade 4		10	100

B, Brunt's criteria.

Table S6. Incidence and multiplicity of PPFs and liver tumors in STAM mice.

Lesion	No. mice	Incidence (%)	Multiplicity (No./mouse)
PPF			
Basophilic	7	7 (100)	2.71 ± 2.43
Eosinophilic	7	6 (85.7)	1.86 ± 1.35
Mixed type	7	6 (85.7)	4.71 ± 5.28
Vacuolated	7	2 (28.6)	2.43 ± 6.00
Total	7	7 (100)	11.71 ± 9.64
HCA	7	7 (100)	6.86 ± 8.67
HCC	7	2 (28.6)	0.43 ± 0.79
Total tumors	7	7 (100)	6.57 ± 8.42

PPF, proliferative preneoplastic foci.
